# "The Hospital" Nursing Mirror

**Published:** 1901-04-27

**Authors:** 


"The Hospital" Nursing Mirror.

Being the Nursing Section of "The Hospital."

[Contributions for this Section of "The Hospital" should be addressed to the Editor, "The Hospital" Nursing Mirror, 28 & 29 Southampton Street Strand, London, W.C.]

### Notes on News from the Nursing World.

### QUEEN VICTORIA'S JUBILEE INSTITUTE FOR NURSES.

The meeting which is to be held at Londonderry House for the purpose of taking steps to organise action throughout the country, in order to secure an adequate endowment for "Queen Victoria's nurses," has been preceded by the presentation of the annual report of the council of the Jubilee Institute "to Her Majesty, the patron of St. Katharine's Royal Hospital," and the supplementary report of the council and the committee of the Queen's Commemoration Fund. We learn from the former that 39 nursing associations were affiliated to the Institute during the year, as against 16 which were removed from the roll of affiliated associations; that the number of nursing associations in affiliation now stands at 484 as compared with 461 in 1899; that the number of inspections made was 886, or nearly 200 more than in 1899; that the number of Queen's nurses has risen from 721 to 763; that the number of nurses trained by the Institute which completed training in 1900 was 155; and that as in 1900 the number of nurses who received certificates on the satisfactory completion of the required term of service was 125. Of the 164 who resigned from various causes, 15 were married, 20 accepted hospital engagements, and 30 took up private nursing. As our readers already know, several new nursing homes were opened in centres where the associations are in affiliation with the Institute, and the report says, "The establishment of these Homes is of importance as witnessing to the permanent hold which nursing the sick poor in their own homes has taken in the large towns and elsewhere, and to the increasing interest which is shown where the nursing work has been set on foot." Mention is made in the supplementary report of the fact that the minimum qualifications as to hospital training for Queen's nurses has been raised to two years, and we have no doubt that ere long the Council will be able to raise it again to three, though, as it is pointed out, the qualifications of very many Queen's nurses are far above the minimum. The statement that "the marvellous growth of the work of mercy which her late Majesty herself inaugurated still necessitates a larger expenditure than the present subscription and donation lists and income derived from the investments of the institute can meet" will serve as a text for some of the speeches on Monday. We are asked to state that any ladies wishing to attend the meeting will be sent a card of invitation on application to the hon. secretaries of the Queen's Nurses' Endowment Fund, 164 Cannon Street, E.C.

### PRINCESS CHRISTIAN'S NURSES.

There are some interesting items in the fourteenth annual report of the committee of Princess Christian's trained nurses. The staff now consists of the lady superintendent, the assistant superintendent, three "Queen's" nurses, one obstetric district nurse, and twenty private nurses, working from the Home at Windsor; and of four "Queen's" nurses in the branch districts of Eton, Chertsey, Addlestone, and Egham. During 1900, the number of cases nursed was 286, and though two extra nurses were employed temporarily, 68 cases were refused. Seven of the nurses received a bonus of £2 or more on their earnings and six private nurses were employed by the Windsor Royal Infirmary committee to nurse the patients brought from the Slough railway accident; the district nurses also assisted on the day of the catastrophe. Two nurses left during the year to be married, two for hospital posts, one on account of her health, and three on the Army Nursing Service Reserve were called up for duty in South Africa and Netley, thus making no less than sixteen past and present nurses belonging to the Home who have served during the present war in connection with the Army Nursing Service Reserve. All these have enjoyed good health, and are still on active service, excepting one invalided home for a short time after enteric fever, another with rheumatism, and a third discharged as required on account of sickness at home. Two district probationers have received training in district nursing, and have been enrolled as "Queen's" nurses.

### CHANGES AT THE NURSES' CO-OPERATION.

We understand that Miss Amy Hughes has this week resigned the post of matron of the Nurses' Co-operation, and that Miss Wells, hitherto matron of the Howard de Walden Home and Club, has been already appointed to fill the vacancy. As these incidents have followed each other so rapidly, we question whether the majority of members are aware that they have occurred. It is evident that internal disorganisation is spreading, and in view of the obvious need of a guiding hand at the helm, we strongly advise members of the Co-operation to insist upon knowing the true state of affairs before it is too late to remedy them.

### THE PRESIDENT OF THE CONGRESS OF NURSES.

It would be interesting to know who was responsible for the absolutely unfounded statement in the *Westminster Gazette* that Mrs. Bedford Fenwick has been elected president of the International Nurses' Congress to be held at Buffalo in September. As we intimated last week, Miss Isabel McIsaac, one of the most popular of American matrons, is both president and chairman of the Congress, but the erroneous announcement has puzzled so many of our readers that we think it necessary to point out that our contemporary was strangely misinformed.

### A NEW DEPARTURE AT CROYDON.

The Croydon Board of Guardians are willing to appoint a qualified medical practitioner as superintendent of nurses. This is a new departure. It will, indeed, be a link between the medical and the nursing professions if a lady is selected to superintend the nurses who may, at a pinch, serve as a deputy assistant to the medical officer. We shall watch the development of this curious innovation with much interest. As a head nurse is also required at the Croydon

Infirmary, we presume that there has been at least one resignation in consequence of the extraordinary manner in which the guardians have treated the matron.

### MISS BOURGUIGNON'S EXPERIENCES.

MISS E. BOURGUIGNON, who was awarded the distinction of the Royal Red Cross by Queen Victoria in January last for her services in nursing the sick and wounded during the siege of Tientsin, sends us some interesting notes of her experiences, which show the severity of the strain she had to meet, and attest her remarkable courage. She writes:

On June 15th the trouble began: they were burning houses all around us, and at 4 o'clock in the morning I was called and told I had to leave the hospital with my patients, and the church bell was ringing to call all people to the Gordon Hall. But as I was further advised that it was not quite needful to quit at once, I remained alone with seven patients till Sunday afternoon. I then had orders to leave immediately, and I removed my patients to the Gordon Hall under fire. It is most wonderful that I was not shot dead in the street, as I had to go backwards and forwards and to walk about under fire for two hours before I could get all the things belonging to my patients. When at last I had them safely in the Hall the wounded kept coming so fast that by the next morning the room they gave us was too small, and we had to remove to the Tientsin Club, where we had more space for them. It was very hard work indeed, as the shelling was so bad and we had very few things to use. It was dreadful to see all my poor wounded men lying under fire, helpless, and to note their sad faces. Most of the people they brought into the hospital were badly injured. We had one gun they called Long Tom, but we all disliked it very much, because when it began to fire it frightened everyone so much and the firing was so heavy. Sometimes the noise suggested the Day of Judgment, and most of the houses were burned and shelled to pieces. It was dangerous to go out as a lot of the Chinese were hiding under cover and sniping the people as they walked along the road. As an example of the severity of the attacks to which we were subjected, I may state that within six minutes I personally saw four shells fall within fifty yards of my position, two of them striking the houses and two raising the dust in the church. Fifty houses at least were struck, so that the absence of casualties was remarkable. One morning just as I had taken breakfast round to all my patients, a shell came in to the club to one of the rooms where my wounded men where lying. I had at that time about eighty patients. The shell took one window right away, all the glass fell in to the rooms and my poor patients lay amongst glass and dust. I could not see them at all for a time, but God was good to us, for the shell did not explode or we should have all been killed that morning. We were working night and day and had no bed or room to call our own, but we were able to keep cheerful and to show a brave heart.

### NURSING AT THE MILITARY HOSPITAL, VALETTA.

THE military hospital at Valetta contains 200 beds. It was not originally intended for a hospital, being at one time a factory. However, at present everything appertaining to the wards and patients is clean, comfortable, and cheerful. There are surgical and medical wards for men, women, and children, an obstetric ward, a very fine operating theatre, and every convenience in the way of hot and cold water. Sometimes, in cases of emergency, English people are taken there, and seem well pleased with the care and treatment they receive. The nursing is undertaken by sisters of charity, who are trained in a nursing institution at Rome. They wear the dress of their religious order, and they are assisted in the wards by male attendants, who also go through a course of training. There are fifteen sisters, including the superintendent, and they undertake day and night duty in turn. The night nursing is done in relays of three at a time: two are on duty up to twelve o'clock midnight, and the third sister comes on about midnight and remains until six o'clock next morning. The men attendants also take night duty—two, or more if necessary, for three or four hours at a time. Neither the men attendants nor the sisters are relieved from day duty as a rule.

### NEWCASTLE NURSES' HOME.

A BRIEF, but highly satisfactory, report has been presented for 1900 by the committee of the Nurses' Home and Training School at Newcastle-upon-Tyne. The institution has now been in existence twenty-nine years, and the staff has grown to one of large dimensions. It consists of 80 trained nurses and 9 probationers. The cases nursed last year were 374 medical, 58 surgical, 48 maternity, 67 fever, 28 nervous diseases, and 20 hospital and infirmary. The silver badge for good service was given to four nurses during the year, and the committee had also the pleasure of dividing amongst the staff bonus money to the substantial amount of £458 10s. An excellent idea of the extensive operations of the Home is afforded by the clear and admirable statement of receipts and payments. The nurses' earnings amounted to upwards of £4,000, the salaries paid to £2,332, the cost of uniforms figuring at £312. There is a balance of £205 in hand of the nurses' sickness fund.

### "SEMI-STARVED" HOSPITAL PROBATIONERS.

A CORRESPONDENCE has been going on in the *Churchwoman* about the alleged hardships of hospital probationers, and the usual number of doubtful stories appear to have been forthcoming. Happily, the attention of Mr. Harry Hems—who has been upon the committee of three hospitals in Exeter for periods varying from twenty-one to thirty-five years, is a munificent supporter of the charities, and knows what he is writing about—was attracted by an attack on the Royal Devon and Exeter Hospital, and he was able from facts within his own knowledge to prove the groundlessness of the accusations. For example, it was said that the nurses of that hospital were "semi-starved," to which Mr. Hems replies that his co-house visitor tried a practical experiment by taking the chair as carver at the nurses' dinner, and he found that his arms were exhausted long before the appetites or the joints. As he adds, this picture does not suggest "semi-starvation." It also coincides with the experience of our own commissioner, who, it will be recollected, visited the Royal Devon and Exeter Hospital last summer, and found that the nurses were treated, as far as he could judge, in a very satisfactory manner, whether as to work, holidays, or diet.

### RECREATION FOR NURSES.

AT a meeting of the Middlesbrough Sanitary Committee last week, the medical officer, Dr. Dingle, recommended the laying out of a tennis court for the nurses. The recommendation was adopted without any manifestations of dissent. We also observe that the Wandsworth Board of Guardians have had the tennis court of the nurses at the Workhouse Infirmary relaid with asphalt. These are pleasant incidents which serve to show that there is an increasing desire on the part of public bodies to recognise the importance of providing suitable recreation for nurses, where the conditions permit.

### BRAVERY NOT APPRECIATED AT WEST HAM.

A SERIOUS accident occurred last week at the West Ham Workhouse, twelve children being injured by the bursting of a water tank. The result would have been still more disastrous if some of the infirmary nurses had not gone to the rescue of the children. The door between the schools and the infirmary being kept locked, they first scaled the wall and then waded knee-deep through water and débris. In fact, as Mr. Chalmers, one of the Guardians, said at a meeting of the Board, they no doubt saved the lives of the children. But a proposal on the part of Mr. Chalmers that the Board should pass "a resolution thanking the nurses for their bravery" was met by protests on the part of the Rev. W. Douglas, who "considered that the nurses had only done their duty," Mr. Keys, who "thought the time had come when they should cease to pander to their officials," and Mr. Good, who objected to members of the Board "toadying to the officials." The resolution was not, therefore, put; and the failure of the West Ham Guardians to entertain gratitude for gallantry stands out in striking contrast with the admirable pluck and unselfishness of the nurses.

### A TRAINING SCHOOL FOR FILIPINO WOMEN.

The first training school for nurses among the Filipino women has been opened at Syracuse, New York State, by Miss Mary Macdonald, who is a graduate of the training school at Bellevue Hospital in New York city, and was appointed as head nurse over a staff of a hundred women in the field hospital of the Seventh Army Corps, at Jacksonville, during the Spanish War. Miss Macdonald was afterwards stationed in Havana with a staff of sixty nurses, and subsequently for more than a year acted as head nurse in the Manila Hospital. Her experiment will naturally be watched with much interest by American nurses.

### OVERWORK AT WEST BROMWICH.

It is very creditable to the two nurses belonging to the West Bromwich Nursing Association that they paid upwards of 12,000 visits last year to 480 cases, but we cannot congratulate anyone else upon the fact. It appears that they were fully occupied the whole of the twelve months. This ought not to be. Nurses imperatively need a holiday. Moreover, if a nurse pays more than a certain number of visits, the patients as well as herself are likely to suffer. The reason why the staff at West Bromwich has not been augmented is that the income will not permit an addition. But, surely, the Association which is collecting funds for the erection of a Nurses' Institute, and expect to complete the building this year, will not find it a very hard task to secure a subscription list of £300, the sum needed. The superintendent not only gives her services free, but assists the organisation financially from time to time. Her generosity should inspire others to emulate it.

### NURSES AS SALESWOMEN.

On Tuesday afternoon if any stray visitors had chanced to look into the garden behind Carnforth Lodge, the home of the Hammersmith and Fulham District Nurses, between three and five, they would have seen tables laid out with all manner of things, both old and new—chiefly the former—eager crowds in front buying, and nurses and friends behind selling. By five only bare tables remained, and a few sad articles which no one wanted at any price—a very prehistoric umbrella, a coat or two that made one warm to look at, and two or three books on army tactics. "We realised over £14" was the news with which one was greeted, at the close of the afternoon's work; a truly wonderful sum when one considers the separate value of the things sold. Evidently, district nurses who are accustomed to turn their hands to almost anything make excellent saleswomen. Not content with this result, one nurse was noticed going off on her evening rounds armed with various things which she knew her district wanted, and of which she hoped to get rid before the day was over. The garden was looking very pretty with its budding fruit trees, and it was, as everyone agreed, so much nicer to have the jumble sale out of doors.

### THE NURSING MOVEMENT IN CORNWALL.

At the annual meeting of the Cornwall County Nursing Association, it was stated that efforts were being made to offer inducements to nurses trained by the Association to work for it after their three years' service by organising a pension scheme in connection with the Royal National Pension Fund for Nurses. This is a step in the right direction, and we are also glad to see that the term for the training of the village nurses has been extended from six to twelve months. Queen's Nurses are employed by the affiliated districts of Helston, Truro, Penzance, Madron, St. Austell, and Torpoint, but the Association has still to depend too largely upon the village nurses.

### PROGRESS AT KILMARNOCK.

The twelfth annual report of the working committee of the Kilmarnock Nursing Association shows that the work of the staff was greater last year than in 1899. The visits made by the nurses exceeded 20,000 in number, each nurse averaging 14 visits per day. The ordinary expenditure was more than covered by the subscriptions and donations, and in addition to the annual donation of two guineas to the Royal National Pension Fund for Nurses the committee contributed four guineas to the Central Home in Edinburgh for the training of nurses. The new home in South Hamilton Street will, it is expected, be ready for occupation next month.

### A NORFOLK CONVALESCENT HOME.

The Children's Convalescent Home at Great Yarmouth has just reopened for the season. The matron is very busy preparing for the reception of thirty-six children from all parts of England. Miss Turner, who was trained at the Jenny Lind Infirmary, Norwich, has been in charge of the home since its foundation seventeen years ago. She is assisted by Nurse Spatchet—who was also trained at the Jenny Lind Infirmary—her faithful colleague for ten years. The home, which is a fine building, is situated close to the best part of the Marine Parade. It is not connected with any Norwich institution, and is entirely supported by voluntary contributions. Miss Turner is always pleased to see visitors who are interested in convalescent work, so perhaps some of the many nurses who choose Yarmouth for their annual holiday, may care to go over the home. If they do so, they will be sure to enjoy their visit and to receive a kindly welcome from the matron.

### SHORT ITEMS.

A very successful sale of work in aid of the Victoria Cottage Hospital, Deal, was held in Deal Town Hall last week, nearly £300 being realised during the two days the bazaar was open.—Miss Edith C. Fry, late matron of Norwood Cottage Hospital, and previously matron of the West Cornwall Miners' and Women's Hospital, Redruth, was married last week to Mr. E. F. Coleman Carpenter, of Beckenham.—Miss Myring, the Army Reserve Sister with whom a representative of the Nursing Mirror had an interview last week, was charge nurse at Huntingdon, not Nottingham, County Hospital.—A Nurses' Hostel will be opened in Manchester on the 1st of June.

### Lectures to Nurses on Medicine and Medical Nursing.

By NORMAN DALTON, M.D. London, F.R.C.P., Physician to King's College Hospital.

LECTURE IX.—CYANOSIS. PIGMENTATION.

*Cyanosis.*—This word means blueness, and it is applied to the diseased condition in which the surface of the body, and the extremities in particular, lose their pinkish appearance and become blue. In health, the blood as it passes through the lungs takes up oxygen from the inspired air, and gets rid of carbonic acid. The oxygen brightens its colour, so that the blood as it leaves the lungs and flows through the arteries and capillaries is bright red. Consequently those parts of the body, where the blood can be seen through the skin, such as the lips, the tips of the fingers, &c., are in health red or pink. In the capillaries the blood loses its oxygen and takes up carbonic acid from the tissues around. The carbonic acid makes the blood purple or dark blue, so that the blood in the veins is dark. Now, if for any reason the blood cannot get rid of its carbonic acid, or cannot take up oxygen in the lungs, it is dark coloured even in the arteries, and all the capillaries contain dark venous blood. Consequently those parts of the body where the blood can be seen through the skin become dark blue. The parts of the body which show the blueness most are (1) those such as the lips, ears, conjunctivæ, nails, &c., which are covered by thin or translucent skin or mucous membrane through which the blood can be seen, and (2) those such as the feet and hands which are furthest from the heart, and in which, therefore, the blood is apt to stagnate and to become heavily charged with carbonic acid.

Cyanosis occurs in all diseases of the lungs or air passages, in which the oxygen cannot reach the blood. It develops very rapidly in acute pneumonia, in obstruction of the windpipe by diphtheritic membrane, &c., and in obstruction of the pulmonary artery by a clot; and it is a very dangerous symptom which is usually treated by the inhalation of oxygen or by bleeding. But it is better observed in chronic cases, such as cases of chronic bronchitis with emphysema and asthma. Patients who suffer from these affections are often stout and their cheeks become marked by a patch of distended veinlets, which can be seen through the skin. These veins are dark red, but if the cheek be exposed to the cold, or if the breathing be further embarrassed (for instance, by exertion), they appear quite blue. The parts mentioned above are also blue, and, in addition, the ears, nose, fingers, and feet are cold and clammy. The patient's breathing is habitually distressed and the pulse is usually quick, small, and weak.

A great degree of cyanosis is also seen in disease of the mitral valve, because the blood stagnates in the capillaries and cannot get freely back to the lungs to be oxygenated. In such cases, the legs are blue, unless they are swollen with dropsy. There is, however, an affection which is called the "blue disease," because it produces cyanosis to such an extreme degree. This disease consists in some congenital malformation of the heart. For instance, a child may be born with the pulmonary artery malformed in such a way that the blood cannot pass freely through it; or with a hole in the partition between the two ventricles or in that between the two auricles. In these cases, not only may the circulation be obstructed, but the venous blood from the right side of the heart becomes mixed with the arterial blood in the left side, so that the blood is dark even in the arteries. As a consequence the lips, ears, &c., become cold and deeply blue, the toes being often quite black. In addition all the above parts become swollen, so that the lips and ears are large, the end of the nose fleshy, and the tips of the fingers expanded so that they form oval swellings like bulbs, for which reason the finger tips are said to be "bulbous." The swelling is not due to dropsy but to the distension of the capillaries and the over-growth of connective tissue.

Children with congenital heart disease usually die young. The age to which they may live will depend on the amount of the malformation, which may be roughly estimated by the extent of the blueness. Still, cases showing well-marked cyanosis and bulbousness of the extremities have been known to live to thirty. There are no special points in the nursing of cyanosis apart from the diseases in which it occurs, but it must be remembered that cyanosed persons feel the cold severely and must be kept extra warm. Also, they are very liable to contract diseases which are predisposed to by exposure to cold, so that they usually die of bronchitis, pneumonia, and consumption. Monthly nurses should know that there is such a condition as congenital heart disease, and if they observe that an infant's hands are unnaturally blue and cold, they should draw the attention of the doctor to the child.

*Pigmentation.*—The dark colour of negroes is due to the presence of minute brown granules in the skin. These are called pigment granules. Even in white people there is a certain amount of pigment in the skin, and the darkness or fairness of the complexion depends on the amount of it. In "Albinos" pigment is practically absent. Pigmentation of the skin may be present in pregnancy, in certain rare skin diseases, in syphilis, and in connection with melanotic (*i.e.* pigmented) tumours, and it forms an essential symptom of Addison's disease. Addison's disease is an affection (usually tubercular) of the suprarenal capsules, and its symptoms are (1) Pigmentation; (2) Progressive asthenia; and (3) Gastric crises like those of pernicious anæmia. Sometimes the last two groups of symptoms occur without the pigmentation. In such cases the occurrence of pigmentation is carefully watched for, and as nurses while washing the patient have good opportunities for observation, they should know what the pigmentation looks like and where to look for it. The discolouration at first looks as if the skin were dirty, but it is soon seen that soap will not remove it. Later on it becomes dark brown and somewhat shiny, so that it has been called "bronzing" of the skin. It never covers the whole body, but occurs in the following places: (1) places which are naturally pigmented, such as the armpits, the nipples, and the neighbourhood of the genital organs; (2) places which are exposed to the air and sun, such as the face and the back of the hands; (3) places which are exposed to pressure or rubbing, such as the shoulders from the rubbing of braces, the waist from the pressure of corsets, &c. Sometimes brown patches occur in the mouth from irritation by the teeth. Any piece of skin which has been blistered or bruised becomes dark brown, but old scars remain white. The pigmented areas, wherever situated, are not sharply defined, but shade off gradually into the colour of the unaffected skin. This discolouration of the skin may be distinguished from jaundice by the fact that it does not cover the whole body, and that the conjunctivæ and urine are not stained.

### To Nurses.

**WE invite contributions from any of our readers, and shall be glad to pay for "Notes on News from the Nursing World," or for articles describing nursing experiences, or dealing with any nursing question from an original point of view. The minimum payment for contributions is 5s., but we welcome interesting contributions of a column, or a page, in length. It may be added that notices of entertainments, presentations, and deaths are not paid for, but, of course, we are always glad to receive them. All rejected manuscripts are returned in due course, and all payments for manuscripts used are made as early as possible after the beginning of each quarter.**

### Across the Seas.

### NURSING AT WINNIPEG GENERAL HOSPITAL.

By a Graduate of 1899.

(*Continued from page* 37.)

The section was divided into three wards, two large ones holding three beds and a cot each, and a smaller one generally kept for a private ward with only one bed in it. There was also a cupboard which was supposed to take the place of a ward kitchen, a bath room, a telephone to communicate with the other sections, and a large corridor in which stood the medicine cupboard, writing table, and doctor's wash-stand. It was small and compact, but as there were more patients than beds, there were one or two stretchers in the large rooms and two of the beds held two children each. All the children—there were twelve of them—were young and fretful. Most of them were Germans, and understood very little English, and as six were on two hour charts, with treatment to correspond, and they fought vigorously at everything I tried to do for them, I did not find much time to worry about my capabilities, and I certainly never worked so hard in my life before, nor found time fly away quite so rapidly. The grown-up patients were convalescent and gave me no trouble.

### Precaution against Contagion.

In these wards we had no hour off duty in the middle of the day, and no afternoon or Sunday, as it was impossible to keep a large enough staff of nurses to relieve in each infectious ward. The rules in regard to preventing contagion were very strict. No "isolated" nurse was allowed to go out of the hospital grounds unless it was for a walk on the prairie, where we ran no risk of meeting any people. Happily the grounds were extensive and beautifully kept, and we had the use of a good tennis court which was laid out quite near the Isolated nurses' home. The next section to mine on the same flat was reserved for scarlet fever, and though the nurse in charge and I were not allowed to visit each other's wards, we often met in the hall and gallery into which both our sections opened, and sympathised with one another's trials and tribulations.

### A Kind Colleague.

The nurse had eleven boys and girls, all convalescent and feeling only too well and frisky: they very much disliked being kept in the hospital for so many weeks after their recovery. Their spirits were very high, and sometimes rose to such a state of exuberance that anyone but a nurse would have succumbed at once to nervous prostration. Except for keeping them in order and daily baths and rubbings with carbolised oil, the nurse was not very busy, and I shall always be grateful to her for the numerous little things which she did to help me at that time. In the general wards it had been the work of the ward maid to keep the refrigerator clean and to polish the brass tap, but in Section D there was no ward maid, and it always seemed to me that the brass tap was the last straw that broke the camel's back. Every morning when I had somehow finished my work about the right time, I would suddenly remember that awful tap on the refrigerator, and rush out to the hall where it stood, with soap and whitening in my hand and wrath and hatred in my heart, and often I found it already polished and bright, and knew who had done it for me, though it was against all rules for a "scarlet fever nurse" to touch a "measles refrigerator."

### A Victim to Measles.

At the end of a month I contracted measles myself, and, to my sorrow and chagrin, I had to go off duty, which was a great blow to me, as in our hospital the nurses were seldom sick, and rather prided themselves on the fact that they were very seldom off duty. I struggled on for a week, feeling desperately ill and not knowing at all what was the matter with me, until one day the house surgeon when going his rounds stopped, looked at me, and said, "Why, nurse, you've got measles! You ought not to be on duty. You wait here a bit, and I'll go and get someone from downstairs to relieve you till another nurse is sent over." However, there was no one that could be spared, and as the lady superintendent was out, I had to remain on duty for an hour or two till she came home, when I was immediately sent to bed in the private ward, where I think I cried for the rest of the day, for I felt so desperately lonely and home-sick. My nurses, however, were kindness itself to me, and the lady superintendent used to come over every day and sit with me for some time. She only visited the infectious wards when one of her nurses was ill, and then she usually came every day. After a week in bed I began to feel better, and determined to be on duty again by the end of a fortnight, that being the time allowed each year for illness, while any time longer had to be made up at the termination of the three years' course. Accordingly I pronounced myself well and capable of going on duty again before I could hardly stand, and felt wretchedly weak; but I also felt that if I had to stay in hospital longer than three years I should certainly die. I was then put on night duty in the same ward. It seems funny to me now to think how I longed for my three years to be up, and yet when I graduated how glad I was to stay on at that dear old hospital as a head nurse, and how sad I was when I did leave it.

### The "Isolated" Home.

After a month and a half of night duty I was transferred to the wards downstairs, which were not so strictly isolated, but were kept for the reception of phthisical patients. Whilst working in these wards we were allowed to go into town and to see any friends outside the hospital, though we were not allowed to go in the general hospital, nor to have any friends to see us in the Isolated Home. We were thus necessarily thrown very much into each other's society, and had to depend entirely on ourselves for amusement. There were usually about twelve nurses in the isolated block at once, and we managed to get plenty of fun, though our material was limited. Our little home was very pretty, containing a fair-sized hall, ten good bedrooms, and a pretty sitting-room, with an open fire-place and a good piano. On autumn evenings we used to gather round the fire, generally with the good intentions of studying "Clara Weeks" or "Isabel Hampton," but it usually ended in making popcorn over the open grate, while we told each other weird and awful experiences which had occurred to us both in hospital and out, but chiefly in.

### A Fancy Dress Party.

About November we felt that we were spoiling for amusement of some kind, so we determined to give a fancy dress party on Hallow E'en. The number of guests was necessarily limited, as the only people who were ever permitted to come to the home were the medical superintendent, the lady superintendent, and the "Isolated" house surgeon; but as they all accepted our invitation with much pleasure we went ahead and spent our spare time in devising new and original fancy costumes out of the very modest material we had at hand. I remember that the diphtheria nurse was a most effective sunrise draped in white sheets which we purloined from the linen cupboard. The best costume was a nun's dress made from shawls and black skirts borrowed from numerous nurses, and the wearer of it ever afterwards went by the name of Sister R—— in the hospital. We managed an elaborate supper which we prepared ourselves, and arranged on small tables in the upstairs hall. The lights were allowed to be kept on for an extra hour, and altogether we had a most successful party.

(*To be continued.*)

### Homes for Inebriates.

By a Matron in Charge.

I suppose that most of my fellow-workers during their nursing careers have had under their care some of those patients termed "inebriates." I do not mean the D.T. cases of one's hospital days, but dipsomaniacs, opium smokers or eaters, and victims to the chloral or morphia habit, not to mention that large and increasing class who will take anything from methylated spirits to eau de cologne, from Cherry Blossom to Trional. We all of us know by experience how difficult such cases are to deal with. The first thing impressed upon you is generally that it must be "kept quiet," thus preventing such precautions being taken as would be effectual, the result being that, from time to time, by some subtle wile or device, the craved-for drug or stimulant is obtained, there is a terrible scene, the friends are heart-broken, and the patient recovers from the ensuing prostration, more degraded, more hopeless, more wanting in strength than ever to battle with the next temptation.

Sometimes under these circumstances, when love and patience alike have seemingly come to an end, the subject is brought up of some "Home" or "Retreat;" but the average nurse who may be in attendance knows little or nothing of such places. Doctors often recommend them, but they are voted hard: English people have a morbid horror of asylums—homes, or any place in fact where it may be necessary for the patient's benefit and ultimate cure to deprive him for a time of complete liberty of action.

They hesitate to send their dear ones where they fancy there will be all kinds of humiliations and degradations for them, trusting to their own care rather; the consequence often being that when they are at length driven by desperation to place them somewhere it is too late. A habit which in the beginning six months' enforced abstinence might have ended has grown to a strength it may take years to eradicate. For the sake of those fellow-workers who care to know something of the workings of such institutions, I will describe the one of which I have taken the temporary matronship.

### No Irritating Rules.

To begin with, there are absolutely no needlessly irritating rules or regulations. Each lady has her own bedroom, and occupies herself as she pleases. She may, subject, of course, to the doctor's approval, attend places of amusement or visit friends if she so desire; but she must not go out unaccompanied, nor must she retain personal possession of money. She also goes to any place of worship she prefers. It is always wisest for patients to enter such homes or retreats under the Act of 1879, otherwise the very fact that things are not fixed, that she can go if she will, the effort of making up and unmaking her mind twenty times a day, and the ensuing suspense and worrying, are apt to make her nervous, unsettled, and irritable.

This is a spacious and comfortable house, and a large enclosed garden behind, where tennis and croquet can be played in the summer, and there is a small lake which in winter, when frozen, provides good skating. A little way off the town with its fine shops proves an attraction for such ladies as are fond of shopping, whilst the surrounding country provides some lovely walks, and rides for those fond of driving or cycling. To afford an idea of how life is passed in such places I will give a sketch of a single day. Breakfast is at nine o'clock, but to that meal few descend, preferring a cup of tea and a biscuit in their own rooms. Yesterday, only two appeared.

### The Inmates.

One is a bright-eyed, sprightly little brunette; her married life had been of the happiest and merriest, and were I but to mention her name there are many who would exclaim, "Why, that girl—she used to be the centre of life and gaiety at Simla and—and—wherever in fact her husband's regiment was quartered!" One day he was killed in one of our little border campaigns, and the poor widow started deadening her grief with stimulant. It says much for the pluck of the little woman that entirely a free agent—her pension secure, and none to say her yea or nay—she, realising how her life was being wrecked, voluntarily placed herself here for a year. The other is a different sort altogether, one of the women who are always right and the rest of the world always wrong. She spends the greater part of her time in alternately writing to lawyers to ask them to take up her case, and bewailing the hardness of heart of her family, the truth being that she has been given chance after chance of retrieving herself, till now, with young daughters at the age to be most humiliated and ashamed of her outbreaks, her long-suffering husband has placed her here for their sakes. At eleven o'clock all gather in the dining-room for some hot milk before going out.

First comes a tall pretty girl of twenty-two. Two causes have brought her here—heredity and the pernicious and fatal habit of always keeping stimulant at hand in her bedroom. Given a not over-strong, highly-strung girl, fond of excitement, the consequent lassitude, and such a pick-me-up always in the wardrobe, and the result may be easily prophesied. Luckily for this girl, her mother was a woman of sound sene and prompt decision, so that to-day an apparently cured girl, she is ready to return to the usual round of life, with no one to know that she has been elsewhere than on a long visit to friends.

### The Advantage of Small Homes.

That is one of the benefits of these small homes. The malady and the means of cure are known to so few that the average patients, when cured, can return to everyday life and its avocations, almost certain that they are not likely ever to run against any of those who knew them in their dark days. Like the prison birds, the "inebriates" must, as far as possible, be started "fair"—raised above themselves and their old ideals, placed where past faults cannot spoil future promise.

### Other Types.

Among other inmates are a dark, passionate, ill-governed, and yet lovable creature, utterly without self-control—it will take some time to effect her cure—one of the feeble-minded folk that are the despair of everyone. Her resolutions are of the best, but she has absolutely no strength of will. Here is a different type. Finding the habit growing—hers is morphia—this woman has deliberately placed herself where, beyond the reach of temptation, cure, however painful, is certain and fairly speedy. Even she, however, fearful of her own power of will, has signed for six months.

Up till eleven o'clock each patient follows whatever bent she pleases, but at that hour it is an unwritten rule that, weather permitting, all should take the fresh air in some form.

To-day two of them decide that they want to go down town shopping, so the doctor's wife kindly accompanies them. The rest think they would like a cycle ride. So we determine on a certain picturesque village some few miles off and get ready to start.

At the last moment one changes her mind, and says she will join the other in the garden, so leaving them under charge of a maid—though with doors with patent locks they are safe enough—we set off gaily for our ride, returning just in time for lunch.

The afternoon turns out wet, and one patient devotes herself to stocking-darning. Another, who is milliner-in-chief to the establishment, alters all our old hats. I go downstairs to make a cake, one patient comes to help and make toffee, and the others play billiards. Tea is served at four o'clock, after which, it having cleared up, we go for a tramp through country lanes. Dinner is at seven o'clock, and the rest of the evening is spent in our usual fashion. That is, one patient plays us Chopin, and sketches from the latest operas in turn; three others and myself play whist; another sits down to collect fresh evidence on her case from some fresh lawyer; and one who is given to altering both her mind and her dress, sits down to carry out the latest and seventh idea concerning one of her frocks.

### THE MATRON'S DIFFICULTIES.

In dealing with such cases, the principal thing seems to be not to leave the patients too much together. In a certain sense such people are degenerates, and what they want is companionship with more wholesome minds, as well as a certain amount of restriction. Of course the lot of matron of such a home is not altogether rosy. There is a great deal of grumbling about everything, from food downwards, or upwards as one happens to view the question. Patients are prone to be nervous, hysterical, and otherwise difficult to deal with. At first they are apt to feel themselves ill-used, and one has to get them to look on their stay here, not as a punishment for past sins, but as a means of restoration to health, to self-respect, and to happiness. Long before the end of their stay they are generally ready to acknowledge this, but just at first they want a good deal of sympathy and patience, and it is not always easy to lift lame dogs over stiles.

### THE NOBILITY OF THE WORK.

Some nurses look down on all such work, but surely it is just as noble to help nurse those with diseased minds and perverted wills back to the ranks of earnest workers as others whose ailments are purely physical. I find I have omitted to speak of the most important factor in the work—the doctor, who with his wife is my mainstay, and the mainstay of us all. He sees all patients daily not necessarily for a private consultation, as often as not dropping in for afternoon tea, and if necessary—which after the first month is seldom—seeing them privately afterwards. He and his family do their best to make things pleasant for those under his care. One evening a party is made up for the theatre, another it is for a concert or lecture, and now the days are lengthening, plans are afoot for photographing expeditions into the surrounding woods and visits to places of interest in the vicinity.

### Nursing at Mooi River Hospital.

### A CHAT WITH AN AUSTRALIAN NURSE.

"I LOVE my work. Nursing the sick brings out the best side of character; it gives a meaning to life," said Miss Croft, when I called upon her the other day to learn something about her experiences as a nurse in Australia and at Mooi River Hospital. It occurred to me that this was a conception of the nursing vocation worth the attention of some of the recent correspondents of the NURSING MIRROR; though we were not discussing the vocation of a nurse at all, but just chatting about where she had been, and what she had done. Miss Croft has done all kinds of nursing; general hospital nursing, fever nursing, private, district, and midwifery nursing, and when the war broke out she wished to experience something of Army nursing as well.

"I gave up my appointment in Melbourne," she said, "and went at my own expense—paddling my own canoe—to Durban. I was not on the Reserve, and I had joined no contingent; consequently I had to wait until the nurses sent by Government, both from England and the Colonies, had received their appointments. I took rooms, and looked about me; and soon I was fortunate enough to be sent to the General Hospital at Mooi River, where I have been ever since; this was in March last year."

### DWELLING IN TENTS.

"You have had a year under canvas?"

"Yes; and I have nothing but the highest praise for the conduct of the hospital; the management is splendid throughout. Miss Cole is superintending sister, and when I arrived there were only ten sisters, and convoys constantly coming in with wounded and sick."

"You worked pretty hard, I expect?"

"Worked? We did work! The tents had not long been up, and there was everything to do. We had just to go in and do what we could. Work like that teaches you to make the most of things and turn everything to account—it brings out the best of your nature. There is no time to worry about trifles, and no time to grumble. We found the orderlies tired out; at work night and day, and with only an hour's sleep snatched when they could get it. Afterwards we got raised floors for the marquees, but at first we had not even these."

"It was a complete change from a civilian hospital?"

"As complete as it could possibly be. An Army Sister has, so to speak, a freer hand; she may do as little as she likes or she may work herself to death. There was plenty to do, only it was of a different kind."

"The life was a healthy one, was it not?"

"Exceedingly so. Tents make splendid wards, for when the sun shines you can fasten them up at the sides, and you can let them down when it rains. It is very comfortable inside."

### THE OUTDOOR UNIFORM.

"The rainy season must be a trying time?"

"Yes, but we went about in mackintoshes and gum shoes, and these, with a straw hat, made up our outdoor uniform. The first six weeks are always trying to newcomers, but after that time, during which I certainly felt ill, I was never sick; never off duty for a day on sick leave."

"May I ask why you joined no contingent?"

"I would not wait to do so. When Ladysmith was relieved we all thought the war would soon be over, and there was great excitement on board among the men; everyone imagined we should be too late. However, it is a year later, and here I am in London, and the war still going on."

### AN AUSTRALIAN HOSPITAL.

"Is it your first voyage home?"

"Yes, my first visit to England."

"Are you visiting any of the hospitals?"

"I should like to see the London Hospital. But I think I should find the management of all very much like what I know of our hospitals in the colonies, and I maintain that at Melbourne Hospital, where I was trained, one gets the finest training possible. It stands first, not in the colony, but in the colonies."

"I should like to hear something about it."

"The hospital is built in the heart of the city, and it receives all cases—medical, surgical, and contagious. The central block has about five hundred beds, and the contagious wing about two hundred. The course is very thorough: first of all strict inquiries are made about the qualifications of every applicant, and she is taken on three months' probation. If unsuitable, she is quietly dropped at the end of that time. The course lasts three years, but many stay four, five, and even six years. The examinations are very stiff; and each nurse is required to go through the whole training in medical, surgical, and contagious wards. It is very hard work, but it is a splendid training."

DISTRICT NURSING IN MELBOURNE.

"I think you said you had also done district nursing?"

"Yes, but I could not bear the strain; unless I were rich enough to help the poor creatures I could not bear to go to them. To think of babies coming into the world with no warmth or comfort, and the kiddies crying for food, is too much for me. The poverty and misery of the garrets and attics in Melbourne is too terrible. I daresay it is worse, and that there is more of it in London, where the population is so much greater; but I feel that that kind of work should be done by those who are able to do it, and I am not."

As I said good-bye, I expressed the hope that Miss Croft's stay in England would be a pleasant one.

"Thank you," she replied; "you see I am, as usual, paddling my own canoe here also. I knew no one on board the *Pinemore* except the sister with whom I came over; but I have always fallen on my feet before, and I have courage to believe I always shall. There is no mistake that I have done so this time, for I have found most kind friends in London, and I am truly grateful for it."

### Nursing in the Erie County Hospital, Buffalo.

By A GRADUATE.

MANY English nurses are taking a great interest in the coming Pan-American Exposition and would probably like to know something about the routine of an American hospital. I am a fully-trained and graduate nurse, received two years' theoretical and practical instruction under the able superintendence of Miss Emma Jane Keating, who, along with Mrs Robb, is first Vice-President of the coming International Congress of Nurses. Miss Keating has held the position of superintendent of the Erie County Hospital, Buffalo, for almost seven years, and has been re-elected for the ensuing four years. The course of instruction used to be for two years, but it has lately been raised to three. The nurses under the two years' system were probationers for the first six months, juniors for the next six months, senior assistants for the first half of second year, and head nurses during the last six months. There is a resident medical superintendent, who has proved his competence by holding that position for a number of years to the complete satisfaction of Democrats and Republicans, neither political party seeing any just cause or reason why he should be deposed. There is also a resident staff of six doctors. In the hospital dining-room are three tables: one very large one occupied by the nurses with their superintendent at the head, a small one for the medical superintendent and his wife, and the third for the use of the resident medical staff. All meals are served at the same time, and discontent in that direction is a thing unknown.

THE COURSE OF INSTRUCTION.

The nurses receive three lectures a week from different members of the large visiting medical staff according to the subject, and each class prepares a lesson weekly, which they recite to the superintendent of nurses, who also gives a practical demonstration before dismissing the class. Bandage classes and the orthography and definition of medical or technical terms are given at the discretion of the superintendent of nurses. During the course of instruction there are at least four examinations. For successful candidates for probationership there is what might be called "an educational test examination" in English subjects, reading, letter-writing, dictation, and arithmetic. At the end of the first year's training pupil-nurses are examined in anatomy, physiology, and class notes. Six months later the senior assistants are examined in materia medica, surgery, and medical nursing. The final examination consists of anatomy, physiology, surgery, materia medica, medical nursing, and obstetrics. After writing up these papers the nurse waits in fear and trembling the announcement of her fate, for if her percentage is below 75 per cent. she receives no diploma. However, failure has as yet been unknown, for incompetent nurses are never allowed to serve the necessary course in the hospital, and the resident medical staff do all in their power to help the nurses in their studies, and are most kind and patient in explaining knotty points till the student-nurse thoroughly grasps the sense and meaning of the apparent mystery, and of course no nurse wants to be the first to bring ignominy on herself and her class; so each and all work and study with right good will, receiving every encouragement from the superintendents.

THE GRADUATE AND THE TRAINED NURSE.

Well, one day the nurse receives many congratulations, and is informed that her examiners expressed utmost satisfaction, and that she is entitled to wear a black velvet band round her cap, seeing which everybody knows that she is a graduate nurse. When engaged in private nursing her inclusive salary is $20 per week. A "trained" nurse in America is a young lady who has no diploma, but has either left the hospital feeling herself incompetent, or has been discharged for the same reason. She is not allowed to wear the black band, nor can she claim the same salary as the graduate nurse. The pleasure of the new decoration is invariably mingled with a note of sadness, for the nurse must leave the hospital which has been to her a happy home for so long; the old familiar sights and faces must be left behind, and she must go forth to make or mar a professional reputation. She knows that many who have graduated before her have already got a firm footing, others again are holding good hospital positions, and a few have joined the fortunes of the country by entering into a contract with Government as Army nurses. There is no reason why she should not be equally successful. So she resolutely faces the future, well assured of sympathy and a never-failing welcome from her *alma mater*, where she can always receive counsel when difficulties arise.

HINTS TO VISITORS.

Here are a few hints to nurses who contemplate visiting the Exhibition and being present at the Congress.

The New York Central is the best and quickest line to Buffalo. The Erie or Grand Trunk has some pretty bits of scenery. Susquehanna country, village, and river are very picturesque, and at Portage the train always slows so that passengers can get a good view of the Falls and surrounding scenery.

The Lehigh Valley route runs through grand scenery, but the company has very little respect for time.

The streets of Buffalo are beautiful for cyclists, and a cycle path from Buffalo to Tonawanda and right on to Niagara Falls is perfectly glorious.

The General Hospital and Medical University are in High Street, the Erie County Hospital in the Main Street, City Lines, about seven miles out. The street car runs to Woodlawn Beach, which is also a nice cycle trip. Athole Springs is a pleasant trip, and for a lake trip Port Colborne is very nice.

Enterprising people wishing to extend their travels will find a very varied choice.

### College Nursing.

By a Trained Nurse.

My first experience of nursing in college was very funny. I was sent for to nurse an undergraduate who was suffering from perityphlitis. I was met by my patient's "bedmaker." The term "bedmaker" is generally contracted in college to "bedder," and denotes a more or less worthy soul whose duties include the cleaning of men's rooms, the lighting of fires, making beds, generally domestic duties, and the carrying home, in a well-known basket under a long cloak, all kinds of sundries which her "men" may or may not want. This particular worthy had only within the last few months been promoted from "help" to "bedmaker." Before taking me to the patient she informed me that he was not a gentleman. Subsequently, I discovered that the reason was that the gentleman (who was a hard-working mathematician) had found it necessary to keep his cupboards locked! My patient was so ill that the doctor ordered him to be kept very quiet, and advised me to secure this by "sporting the door" to prevent kind undergraduate friends from rushing in to inquire how he was. Not having completed my education in University slang I was somewhat hazy as to the meaning of "sporting the door," and much to my patient's amusement began to support his inner door from the inside by barricading it with a large sofa! In college rooms the only way to secure quiet is to keep one's oak sported.

### The Difficulties of Nursing.

In cases of sickness happening in college rooms (which fortunately is not very frequent), nursing has to be done under difficulties. One finds very few things for the nurse's use in a man's room. If poultices are ordered, one may have to keep the linseed in the patient's flower-pot or his tobacco-jar. However, everybody is good in lending things for patient and nurse. The bedmaker brings blankets, rugs, and other useful things which she may find in other men's rooms, with or without their owner's permission. I have constantly heard voices shouting down the staircase, "Where's my pillow; who has got my blankets," &c., to be answered by the worthy bedder, "I've just lent them to Mr. ——'s nurse, sir." The owner then retires, and cheerfully goes without his property until it is returned. I have always found undergraduates most delightful patients to nurse, and very grateful for anything one may do for them. In cases of illness in college it is most necessary for them to have a trained nurse, as they are alone in their rooms, away from home, with no one to take care of them excepting their bedmaker, who is only in college during the day. The undergraduate is a loyal creature to his fellow-undergraduate, and always most kind to a nurse, though he seems unable to grasp the fact that a patient cannot be left. For instance, one will come cheerily in, saying, "Nurse, I've heard it is very bad for nurses to have meals in the sick-room, come up to breakfast with me;" or "Nurse, I've ordered a dog-cart; I've heard it's so good for nurses to get fresh air; will you come for a drive?" Needless to say I could not accept these kind offers, although I thoroughly appreciated them.

### An Influenza Patient.

On another occasion I was sent to a patient suffering from "influenza," he was not ill enough to need a nurse to sit up with him all night, so the physician advised me to go to bed, and come in occasionally to see whether he needed anything. On entering his room at midnight, thinking he might require a little nourishment, I found three other men playing cards with him! On expressing my astonishment and gently hinting that this was not the kind of thing for an invalid, they quietly left, and all called the next day to apologise to me. On remonstrating with my patient, he explained that he was feeling so lonely that, hearing some friends tapping at his window, he had admitted them rather than disturb his nurse. My college nursing experiences have taught me that one must not rely too implicitly on the truth of college stories told by patients. I remember a man telling me that for some time he had, as a great honour, to sit next to the Dean in Chapel; I found afterwards that such honours were only conferred upon men whose conduct there was not beyond reproach. Another told me how celebrated his college was for its buttered toast and ketcheree. I was foolish enough to believe this, and when nursing a Professor in the same college later on I referred to these things as being so famous, he replied, with intense amusement, that it was the first time he had heard of it.

### Sponging Between Blankets.

Another patient was an undergraduate suffering from pneumonia and pleurisy. After the physician had seen the patient and had written the prescription for his medicine, which I was to get dispensed at once, I sent one of two undergraduates, who were waiting to see if they could do anything, to fetch the medicine, which it was important that the patient should have as soon as possible. The willing messenger fulfilled his commission with great dispatch, only arriving to drop the precious bottle upon the mat and to spill the whole of its contents. Notwithstanding this catastrophe the patient made a good recovery. During his illness it was necessary whenever the temperature rose above a certain height to sponge him between blankets, as only a nurse can do. Truly "a little knowledge is a dangerous thing," for the day after I left, the patient's mother, who was staying with him, suggested that he should remain in bed while she tried the experiment, as, in case he should require the same treatment at any future time, she would like to be able to do it. Accordingly, being a dutiful son, he submitted, but he evidently did not enjoy the process or its results, for he wrote the next day to tell me he had had to sit in front of the fire while the blankets were dried.

### A Poultice in the Wrong Place.

In the case of another patient the doctor who had been called in had told the landlady to put a linseed poultice on his chest, to be renewed when the nurse should arrive. On entering the patient's room I found him looking very depressed and in great discomfort. When I went to renew the poultice I found that the instructions "To put a poultice on his chest" had certainly been carried out, but on the wrong side of the night-shirt, the poultice about an inch in thickness. He had intimated to the landlady that he could not bear it hot, so she had put it on outside the night-shirt. However, when a properly made, thin and hot poultice was applied next to the skin he thoroughly appreciated it.

### Behind the Curtain.

On another occasion an undergraduate patient kindly said to the bedder "Take care of Nurse." She proceeded to do this by ordering in bottles of stout, which she herself consumed and put down to me, evidently thinking it was necessary to keep up her own strength in order to carry out his injunction. When the patient was quite well he suggested that on the last evening before I left him he should invite two or three men in to sing. Accordingly, at about eight o'clock, instead of "two or three men," about ten arrived. As the patient had quite recovered there was no fear of the excitement proving too much for him; therefore I saw no reason to forbid it. After coffee and light refreshments each man sang a very good song, such as "Cleansing Fires," "For all Eternity," and the "Promise of Rest." This, however, was too good to last; and I found that they had saved as "a grand finale" Corney Grain's well-known song with a ringing refrain, "He did, but he didn't know why." When the lively strains of this chorus reached the ears of the Dean, whose rooms were in the same court, he sent the college porter to find out who was so thoughtless as to be making such a noise on a staircase where there was an invalid, besides other men who wanted to be studying. The porter ascertaining that the noise was not from any other room on the staircase, came to the rooms of the invalid, and discovered that the concert was taking place there. I had to stand behind a curtain while the Dean's message was delivered! Then, after "God Save the Queen" had been softly sung, with a cheer for nurses in general, the party quietly dispersed. The next day the Dean said to the patient, with reference to the concert, "How different it would have been had his nurse been there!"

### Nurse's Literature.

Next I nursed an undergraduate who was very dangerously ill, and who was living in rooms kept by a landlady of the "superior order." She had been the wife of a highly respected college porter, and an enthusiastic follower of the hounds, who, with his steed, are now deceased. She was always telling me of the better days she had seen, and made it most difficult for me to get anything done for my patient or his people, as she considered everything beneath her dignity. This was a case in which the physicians thought that recovery was almost impossible. However, the patient, who had been so near to the brink of the grave, recovered. He was the son of a very popular "hunting squire," and enjoyed all papers containing news of sport, amongst others one I had never seen before, called the "Pink 'Un." One day when the physician called this paper was lying on the bed, he at once said, "Are you well enough to be reading the sporting news?" and this naughty boy answered, "I had it brought up on purpose for nurse, as she is so fond of it!"

### The Story of a Professor.

Besides these younger members of their Alma Mater, I have also had the privilege of nursing a great Professor of History, a Professor of Botany, a Professor of Mathematics, a great Theologian, a great Physician, and a great Classic. I will mention one story connected with a Professor whom I was nursing. He had broken down through overwork, was very seriously ill, and was ordered perfect rest of mind and body. Three physicians had called, and all had said, "Professor, you must not use your head." To one well-known physician he had answered, "My head is my own, I shall do as I like with it." No sooner had the door shut on the medical men, than, in a tone of authority he said, "Nurse, I don't care for any doctor, I command you to get my papers," evidently thinking I should get them at once, for he seemed much astonished when I said, "You will have to command all night, Professor, and you will not get them." After waiting a few minutes he asked me for the second time if I intended to bring him his pen and papers, and on my again answering in the negative, he just managed to get his legs out of bed, probably thinking that I would imagine that he was going to try and get them himself, and to prevent him doing this, would obey his command. On finding that I really meant not to get them, he consoled himself by saying, "Ah! trained nurses are a great bore, but I suppose I must get into bed." He certainly could not have cherished any ill will, for when he was again ill he at once sent for me. There is so much of pathos, so much that is sad in nursing, that I think seeing the humourous side of life, as welll as the necessarily sad part, helps to keep one cheerful and bright.

### Appointments.

Bradford Royal Infirmary.—Miss Annie Broadbent has been appointed night superintendent. She was trained at Bradford Royal Infirmary, and has since been attached to the Bradford Private Nursing Institution.

Dumfries and Galloway Royal Infirmary.—Miss Jessie Calder has been appointed lady superintendent. She was trained at the Edinburgh Royal Infirmary, and has since been matron at Dufftown, matron at Coldstream, and matron at Chalmers's Hospital, Banff.

Grantham Hospital.—Miss Minnie Morrison has been appointed sister. She was trained at the Arbroath Infirmary, and has since been staff nurse at the Royal Hospital for Sick Children, Edinburgh.

Newport (Monmouthshire) Union Infirmary.—Miss May Catherine Jones has been appointed superintendent nurse. She was trained at the Kent and Canterbury Hospital, and has since been staff nurse at the Sussex County Hospital.

Sevenoaks Union Workhouse Infirmary.—Miss Catharine Rhoda Emily Skoines has been appointed superintendent nurse. She was trained at St. Pancras Infirmary, where she was afterwards assistant nurse. She has also been charge nurse at Romford Infirmary, superintendent nurse at Northampton Infirmary, and superintendent nurse at the Workhouse Infirmary, Plymouth.

St. George's Union Infirmary, Fulham Road.—Miss Alice Wells and Miss Rose Bissell have been appointed sisters. They were both of them trained at St. George's Union Infirmary for three years.

### Presentations.

Aberdeen East Poorhouse.—A meeting of the officials of the East Poorhouse, Aberdeen, was held last Saturday to bid farewell to Nurse Armstrong, who is leaving for Edinburgh, and Mrs. Main, who has obtained an appointment in a London hospital. After tea, Mr. Dalgleish, the governor, who presided, presented Nurse Armstrong, in name of the members of the staff, with a beautiful dressing-case, at the same time expressing high appreciation of her admirable services as a nurse in the East Poorhouse. He then handed over to Mrs. Main a silver chain and cross—also a gift from the staff in recognition of capable and devoted service. The Rev. A. Mackay, chaplain, returned thanks for the ladies, assuring the donors that the mementoes would be greatly prized by the recipients.

### TRAVEL NOTES AND QUERIES.

The Italian Lakes (Nomad).—Most certainly I should consider July too late; it can be very hot on the lakes and you feel the heat. May is the month of all others, but if that cannot be arranged why not come home so as to be in Como at the end of September or beginning of October. It is difficult to offer an opinion of their relative merits: personally, I think I give the preference to Como and Garda.

South Brittany for Ten Days (Stella).—I am sure you would find it a mistake to go so far for such a short time. The distance is not so very great, but the time occupied in getting there is out of all proportion—it takes almost as long as to get to Milan. The trains to Southern Brittany are not very frequent, and should you miss the early morning one your position would be very vexatious. Will you entertain this idea? All is new to you there, why not cross on Friday night to St. Malo, go on to Mont St. Michel, seeing the pig market at Dol on Saturday morning, leave the Mount early, and spend two nights at Dinan, from there to St. Briene where take diligence which meet each other round the splendid North Coast, sleeping at Paimpol and Lannion; from there train to Morlaix from whence you can visit Roscoff and St. Pol-de-Lehon, and then home.

A Fortnight Abroad (Dagmar).—I should recommend a little tour on the Loire, or in the Ardennes, or, if you prefer the sea, you could see a good deal of Brittany in a clear fortnight; then there is another alternative—a quiet stay in one of the small Normandy sea-coast towns like St. Valery-en-Caux or Veules. If you will write me again, to which of these plans you incline and how much you wish to spend, I will draw out a tour for you. The Loire trip is chiefly attractive from the many historic châteaux to be visited along its banks.

Rome in the Summer (Glauca).—It would be very unwise to remain there (unacclimatised as you would be) through the hot months, and why wish to do so when it is so easy to leave there at the end of May and go to the Tyrol or to Switzerland, returning to Rome in October. Moreover, the artistic effects are not so good in the summer, because the vegetation which clothes the hills is in the summer so rich that the amethystine hues which were so lovely in winter have entirely disappeared. November and December are the best months for sketching; the atmospheric effects are splendid, and it is not so cold and wet as January and February.

Holland for a Cheap Holiday (C. E. B.).—You give no pseudonym, but I hope this will catch your eye. I do not think you have read the articles in the Nursing Mirror for February 2nd, February 16th, and March 2nd. In February 2nd the question of expense is dealt with. Holland is not a cheap country, partly because the competition is not great in hotels and partly because the coinage is not so much to our advantage. Tell me what you can spend and I will give you a list of hotels.

### East London Nurses at the Mansion House.

There was a good attendance of friends and supporters of the East London Nursing Society at the Mansion House on Tuesday, and, as usual, the matrons and nurses were present in uniform. The Lord Mayor presided, and the Lady Mayoress was also there. The Lord Mayor very heartily welcomed those present to the Mansion House, and said that the Lady Mayoress particularly wished to join with him in doing so. "We believe," he said, "that the society is doing a very good and useful work." In case there were any who were not thoroughly well acquainted with the objects of the society, he explained that it was established "to nurse the sick poor of East London in their own homes by means of trained resident nurses." It would be at once patent to all that such an object could not fail to ensure their esteem and support; the cause needed no advocacy, for it spoke for itself—"skilled nursing for the poor of the East End." He referred to the work that was being done. "The society was established in 1868, and he learnt from the report that 5,028 cases had been nursed; of these 782 were men who had thus been helped to regain strength, and with it the power of maintaining themselves and their families. He took it for granted that all these cases were drawn from the working-classes; therefore, if by skilled nursing it had been possible, under the providence of God, to restore 782 men to health and wage-earning capacity, it was a very great proof of the amount of excellent work being done. It was work of the very highest character."

### The Bishop of Kensington on Nursing.

Then the Bishop of Kensington proposed that the report be adopted, and the committees re-elected. He said he was glad to help, in ever so small a way, such an excellent society. He welcomed any link that would knit together the East and the West; and it was one of the elementary duties of bishops to promote a real spirit of harmony. The romantic idea that all the riches were in the West and all the poverty in the East was one which he would gladly help to dispel by acting as guide to parts of London usually called wealthy. He was all the more pleased to speak for the East London Nursing Society, because East London was, for a very short interval, without a bishop of its own. A public meeting had two objects: first to diffuse information, and secondly to rouse enthusiasm; and he could imagine no object more likely to rouse enthusiasm than this society. One good thing about it was its exceedingly broad platform; it was one on which everyone could stand; there was no room for the controversial element which could not always be kept out of other societies. Another point was that it was so exquisitely human. What was human appealed to us more than anything else, and, more than anything, the spectacle of human suffering appealed to us. The rich knew what it was, perhaps in the time of an epidemic, to rush about in hansoms, and send many telegrams, in the effort to find a trained nurse; how, when she came, the whole aspect of affairs changed; there was quiet and order; "but," the Bishop went on, "in order to know what it is for a poor man to be ill, you must have a baby crying all day, a wife going out to earn bread, and your wounds untended. I feel so sure that must appeal to you that I need hardly do more than mention it, and leave it to your sympathy. We want not only to help, but to help the right people in the right way. We want also to avoid duplication of agencies. A glance at the report would show that help had been given in cases where help was required, and could not otherwise be obtained; and the society was most inadequately supplied with funds for doing this. Its income was not more than £2,700, and too much of this was raised by entertainments, which were a precarious source of income. More annual subscribers were urgently needed. If help were not forthcoming, it meant either that people were ignorant of the need, or indifferent to suffering; and no place had done more to prove that this was not the case than the Mansion House."

### A Professional Opinion.

Dr. T. Gilbart-Smith, of the London Hospital staff, seconded. He spoke in the highest terms of the nurses' work, with which he was very well acquainted. His sympathies were with the women and children, and he was not sure that in many cases "the woman was not a better man than the man"—at any rate, she was frequently the bread-winner. "It is a glorious work," the speaker went on; "it deals with humanity in its various phases. That's why we physicians and surgeons love our work; because it brings us face to face with human needs, with human good and evil, and gives us an insight into the nature of things as we believe no other profession does. And when we are aided by such women as we see here we can but be proud of the success gained. A countryman of his, when visiting the Falls of Niagara, in reply to the remark of an American, 'Isn't it a wonderful sight?' had said he didn't think it was at all so wonderful; 'what was to hinder it?' He hoped that after such a meeting as this, nothing would hinder this society from having the sinews of war provided, so that its good work might be continued. The gratitude of the poor patients was remarkable, as might be seen by the way they collected pennies to help on the work. The London Hospital was watching the society's work with an intelligent eye."

### Other Speeches.

After the report had been adopted, Major W. Evans-Gordon, M.P. for Stepney, made an eloquent appeal for funds. The founder and the founder's sister were present, and it seemed, he thought, almost presumptuous for such a newcomer to speak on the society's work. He referred to the presence of the press representatives; it was on their kind voice that he relied for making known the wants of the society, so that the urgently-needed funds might come in. "If good nursing was a blessing to the rich, how much greater to the poor? In the rich house sickness meant additional luxuries, straw laid down in the street, and the most complicated scientific apparatus. To the poor it meant that just at the time when extra money was wanted, most probably the capacity to earn it disappeared; one by one the household goods found the way to the pawnshop, and even the bedding might have to go. The nurse almost invariably gained the confidence of those she visited, and her visits meant much more than technical nursing merely. The hospitals could not reserve beds for cases that required only good nursing, and it was to such as these, and to the chronic cases, that the East London nurses went. No branch of science had made such strides as medicine and surgery, and the nursing profession had gone hand in hand with them; the work of the most skilled doctors would be inadequate if unaided by the skilled nurse. There was no society to which he would sooner trust money than this."

Dr. T. H. Arnold Chaplin claimed to speak with some show of authority, for he had been in close touch with the work of the society in the East End; it was in the van of the good agencies in East London. The district nurse worked under different conditions to those of a hospital ward. The quieter footsteps of the district nurse had been almost unheard amidst the tramp of soldiers during the last two years; the half-articulate cry of outcast London was for more help. The work was unostentatious, but it spoke for itself: "good wine needeth no bush."

A very hearty vote of thanks to the Lord Mayor brought this very successful meeting to a close.

### Echoes from the Outside World.

### AN OPEN LETTER TO A HOSPITAL NURSE.

THE publication of the war honours is especially welcome even to those who have no particular interest in the rewards, because it amply confirms the hope which has already begun to dawn that the war is coming to an end at last. The general opinion amongst those who have studied the question is that long as it seems it is really a very short list considering the length of the war and the hard work and severe experience which most of the men have undergone. Lord Kitchener is made a G.C.M.G. (Grand Cross of St. Michael and St. George), and the same honour falls to Sir George White and Sir Redvers Buller. Lord Kitchener gains many places in rank by being antedated as a lieutenant-general, and it is felt that something special will be given to him when he returns after having settled up affairs in South Africa. Therefore his present reward does not seem so inadequate as that bestowed on Sir Redvers Buller, who in the ordinary course of events has finished his work in South Africa, and is therefore entitled at once to such honour as the Government may think is due to him. He was made a G.C.B. (Grand Cross of the Bath) as long ago as 1894, therefore it was impossible to give him less than the G.C.M.G., and considering that for a long time he bore the whole burden of responsibility in South Africa, that he was actively engaged for a whole year, and that he was the reliever of Ladysmith, his friends and his admirers (and amongst the men who served under him he has many) naturally feel that for some reason he has been given as little as possible. It is said that though Lord Roberts in his last despatch spoke of him very coldly, and refrained from praise, notwithstanding that he gave much eulogy to many others, he has since striven hard to secure more substantial recognition for Sir Redvers from the Government, but in vain. The fact that Major-General Baden-Powell has only been given a C.B. has also occasioned much wonderment, and most folks had believed that General French, who has seen such long service and been so extraordinarily successful in all he has undertaken, would have received higher honour than the K.C.B. which has fallen to his lot together with Lord Methuen, General Pole-Carew, and General Hector Macdonald. Still, whatever had been done someone would have grumbled, and it is a matter for congratulation that the South African soldiers, Generals Brabant and Dartnell, have not been overlooked, that the Canadians and Australians have been well remembered, and that even the dead, for the satisfaction of their mourning relatives, have been specially mentioned when deeds of daring had entitled them to the Victoria Cross or the Distinguished Service Order.

---

THE method by which Sir Michael Hicks-Beach intends to raise the £55,000,000 required to pay the expenses of the war for this year was announced last week. The subject is not particularly interesting to nurses, as only a limited number are householders, but still they like to know the history of their country as it is formed. Though only £41,000,000 is required for immediate use £60,000,000 is to be raised by borrowing, and, in fact, the sum offered to the public was subscribed many times over, which suggests that increased taxation might have been dispensed with. As it is, there is ½d. per pound put upon sugar, 1s. a ton upon exported coal, and 2d. more upon the income tax, making it 1s. 2d. in the £. There has already been a tremendous outcry against the enormity of compelling the poor to pay ½d. a pound extra for sugar, and of reducing the profits of the colliery proprietors, but comparatively few protests from the middle classes who have principally to bear the increase in the income tax. I fear that until they form themselves into some sort of a Protection Society, and give up their apathetic resignation, the Chancellor of the Exchequer of the day, will continue to make them bear the burden of any extra expense which may be incurred by the country.

---

THE New Gallery was opened to the public on Monday. There is no single picture which is likely to stand out conspicuously from the others, but for all that the exhibition is a very pleasant one, and is especially strong in portraits. Amongst those deserving of the highest praise are the works of Mr. J. J. Shannon. He contributes "Lady Carbery and her Children" and "A Portrait Group"—the latter, it is of interest to note, representing the painter, his wife, and daughter. The portraits are said to be life-like representations, and were probably a real labour of love. "Lady Carbery and her Children" is very prettily grouped. The boy stands apart, but the little girl nestles lovingly and sweetly against her mother, who is still young and very charming. Mr. Sargent has painted "The Duke of Portland" with his collie dogs—an excellent, manly portrait, but possessing no special feature. His other canvas represents "Mrs. Garrett Anderson, M.D.", and there is much that is fine in the picture, but the painter has unfortunately accentuated the controversial expression—if I can use such a word—of the lady's face, which is a pity, because those points for which Mrs. Garrett Anderson fought and contended are now facts acknowledged by everyone, and she might more fitly have been represented resting calmly and grandly on her laurels.

---

AMONGST pictures which are not portraits are a group of three by Mr. Watts. Two are allegorical—"The Slumber of the Ages" and "Greed and Labour"—the latter showing how the God of Money seeks to lure the young labourer away from the healthy life of his country fields. The third is particularly delightful. It is called "Trifles Light as Air"—a number of cherubs or cupids floating about in space, able to support themselves, happily, in the most ethereal if the most beautiful, of atmospheres. "The Bathers," by Lady Stanley, will easily claim notice, so also "The Casket Scene from *The Merchant of Venice*," which is painted with all the wealth of colour and the skill in manipulating rich fabrics which have made Sir J. D. Linton famous. Mr. Pickering's "The Day's Requiem, Argyllshire" shows the master hand of one who loves the Scotch mountains, whilst Mr. Austen Brown has gone to Holland for his "Sunshine and Shadow." The scene is just a Dutch mother and her children, set against a background of river and red roofs, but it is a picture one would like to hang upon the walls of one's room and gaze upon at leisure for rest and refreshment.

---

RATHER an interesting case was tried last week, in which the proprietor of some residential flats sued a late tenant for a quarter's rent of her rooms. She had left, owing to discomfort, within a month of taking possession, and had paid for her rooms at the rate of £250 a year, 14s. a week for servants, and for such meals as she wanted at tariff rates. When she made her arrangements she was assured of a continuous hot-water service, and was informed that there was a passenger lift in charge of a porter, and that the service was good. All these assurances influenced her considerably in her decision to become a tenant in Harewood Place. When the tenant came into residence she found the hot water supply so defective that she only once had a hot bath all the time; the "passenger" lift was also used for coals which accompanied lady visitors in their ascent, and was worked by a dirty boy in a dirty flannel shirt and no collar; and the service was distinctly slovenly. The plaintiff, though admitting the failure in the hot water service, seemed to think the other matters very trivial, and maintained that the defendant "did not exercise sufficient patience." Mr. Justice Mathew evidently did not agree with him, for he ruled in favour of the defendant with costs. I am half afraid that if the tenant had been a lady of limited income, and the flats bringing in only a low rental, the result might not have been the same; but there is always a distinct advantage in such decisions being given, because they establish precedents and enable clever barristers to quote a case in point when required.

### Everybody's Opinion.

[Correspondence on all subjects is invited, but we cannot in any way be responsible for the opinions expressed by our correspondents. No communication can be entertained if the name and address of the correspondent is not given, as a guarantee of good faith but not necessarily for publication, or unless one side of the paper only is written on.]

### MISS FOX'S LECTURES.

"A. J. K. HAMMERSMITH" writes: I wish to thank Miss Margaret Fox, matron of Tottenham Hospital, for her very instructive and deeply interesting lectures to head sisters.

### MALE NURSE.

"R. J. R." writes: A "Male Nurse" writes in your last issue: "Is there a book which explains the use of a hypodermic syringe?" Now does this question not look absurd for a male nurse (so termed) of 4½ years' experience to ask? I say yes, very. It is such questions appearing in print which lower the public estimation of male nurses. I wonder what "Male Nurse" wants to know regarding use of syringe. Has he had 4½ years' experience and never used a hypodermic syringe? If so, I am afraid his experience amounts to very little. Again, would it not look much better for one who has not had a course of training in nursing to term himself "male attendant" or attendant, and not male nurse? For no one can be a nurse unless having received a course of training and be certified as such.

### TWO CONCEPTIONS OF THE NURSING VOCATION.

"A FRENCH WITNESS" writes: One of your correspondents has described nuns as "unsexed nonentities." Ten years' experience of a nun who attended hospitals has led me to consider them rather as hypersexed somebodies! There is a great deal of untrue poetry going about concerning nuns, started by people who have no notion of the real duties of a nurse. I have seen nuns stoutly refusing to help when but mere boys under twelve years were operated upon for hernia, and other cases which suppressed the wearing of a shirt. I have daily seen them allowing young women to go and be douched, &c., by a student, and alone with him too. I have known a case of hernia which had an almost fatal ending through the nun refusing to give a bed-pan to a boy of sixteen. Many hours elapsed before the male attendant came in, and then all the dressing was soiled and the wound infected. I have scolded nuns for advising their patients to hush up some of their ailments for the sake of modesty. Nuns would not give men a blanket bath even if they knew how to, which they don't. It has also been said that nuns are self-sacrificing. I cannot agree with that either, as I see them daily sacrificing the patients to their own rule and ways, and yet sticking hard to the hospitals and asylums, where they manage to get wealthy through the poor. In France the Little Sisters of the Poor own household property valued at 27 millions of francs, and the Sisters of St. Vincent de Paul property valued at 64 millions. As to their real wealth it is impossible to tell what it may amount to. Therefore nuns cannot be called unsexed and self-sacrificing.

### THE CROYDON CRISIS.

"JUSTICE" writes: I am glad to see expressed by "Deeply Interested" the feelings which I believe all conscientious Poor Law infirmary matrons must have with regard to the principle so nobly defended by Miss Julian. Anyone who offers herself for the post of superintendent of nurses under the Croydon Board of Guardians under present conditions ought to be looked upon by the nursing profession as a traitor to its best interests. Some, no doubt, will apply—unprincipled women who care only for the salary, and with the hope of ultimately stepping into Miss Julian's place. She, I hope, will keep where she is till the Local Government Board express their disapproval of conscientious signing of certificates by dismissing her after so many years of faithful service to the Board, and state plainly their reason for such dismissal. Their action is absurd. If Miss Julian has not merited dismissal, why is she not still to fulfil all the duties of the post to which she was appointed, and for which she draws her salary? And why are the ratepayers to be put to the expense of a salary of £75 to £100 per annum for an extra officer if Miss Julian be fit and capable to retain the post which she has ably filled for so many years? For it does not appear that inefficiency on her part has ever been reported to the L.G.B. I, as the former matron of a large Poor Law infirmary, know by experience how impossible it is to weed out unsuitable nurses. The medical officer would not agree to the reporting of nurses even for faults regarding morality for fear he should be "laughed at" or get disliked; then it was useless my reporting them alone, as he would defend them. This is only one of the results of the wrong principle of allowing medical officers, no matter how young, the direct charge of a number of women instead of the matron being the responsible head of the female staff and all reports respecting their faults connected with the medical officer's department being reported by him to her. Until this is done we shall never see smooth working or the best class of women as nurses in Poor Law infirmaries. Miss Julian has my heartiest sympathy.

### THE NURSES' CO-OPERATION.

"APRONS" writes: It seems very strange that the Howard de Walden Home should be expensive and yet not pay, because it was partly a gift. The committee led the nurses to understand that the Home would be a good investment for their earnings; but is it? Where do the advantages come in to those whose money has been put into it? My earnings from the first have been sunk in it, yet I have to pay 5s. a year towards it, and if I use it to reside in it costs me 30s. a week to be comfortable—as much as I should have to pay in a West End boarding house which is run for gain. "A Mental Nurse" says in her letter in the MIRROR of April 13th that though the Home is expensive, "we are the gainers at the end of the year!" How can we be, when the Home was run at a loss at the end of the year, as the report shows? For my part, I cannot afford to live between my cases at the Home, and there must be many other nurses like me; and so, surely, the Co-operation nurses are still without what they want, for their Home is more expensive than those that are run for gain, although it has been helped by Lady Howard de Walden's generosity. Surely we ought to question whether we have a really business-like committee capable of managing our affairs for us. The letter of "A Mental Nurse" also shows why we are so helpless and ignorant in regard to our Co-operation. She says we do not know the members, and we do not send our voting papers back, which is quite true. Of course this is a great mistake, but what can we do? We are most of us continuously busy with our cases, and many like myself know little of the Co-operation now, as we have our own connection, and only continue to belong to the Co-operation from feelings of loyalty. I know several nurses who are as little satisfied as I am, but these have said to me that if they complained to the committee, they would be made to suffer personally. It is difficult to get anything done when people have this feeling. I fear it is true that we are not public spirited, as Miss Morten complained in her letter on March 30th. Still, amongst so many nurses, we ought to find enough ready to try to get a really good committee, if only we knew how to set about it. Those who founded the Co-operation gave a great deal of time and trouble to it, and many of the nurses showed themselves brave and unselfish in risking their money in the experiment, and it would be a great pity if after all this harm came to the association. It ought to be as well managed as possible, so that nurses could feel safe in joining it. Besides which there are many private adventurers in nursing homes, who would be only too glad to prove that nurses cannot manage a co-operation, and in this way a lot of harm would be done. The blind contentment "A Mental Nurse" seems to advise will not shut the eyes of the public if our management is bad, nor help us to correct mistakes.

### For Reading to the Sick.

"THOU RENEWEST THE FACE OF THE EARTH."

GALES from Heaven, if so He will,
Sweeter melodies can wake
On the lonely mountain rill
Than the meeting waters make.
Who hath the Father and the Son,
May be left but not alone.

Sick or healthful, slave or free,
Wealthy, or despised and poor—
What is that to him or thee,
So his love to Christ endure?
When the shore is won at last,
Who will count the billows past?—*Keble.*

O Earth,
I count the praises thou art worth,
By thy valleys warm and green,
By the copse's elms between,
By their birds which, like a sprite,
Scattered by a strong delight
Into fragments musical,
Stir and sing in every bush;
By thy silver founts that fall,
As if to entice the stars at night
To thine heart; by grass and rush,
And little weeds the children pull,
Mistook for flowers!

. . . . .

Praisèd be the mosses soft
In thy forest pathways oft,
And the thorns, which make us think
Of the thornless river-brink
Where the ransomed tread:
Praisèd be thy sunny gleams.
And the storm, that worketh dreams
Of calm unfinished:
Praisèd be thine active days,
And thy night-time's solemn need,
When in God's dear book we read
*No night shall be therein:*
*E. B. Browning.*

What inexpressible joy for me to look up through the blossoms and the fluttering leaves and to see God's love there; to listen to the thrush that has built his nest among them, and to feel God's love, Who cares for the birds, in every note that swells his little throat; to look beyond to the bright blue depths of the sky, and feel they are a canopy of blessing—the roof of the house of my Father; that if clouds pass over it, it is the unchangeable light they veil; that, even when the day itself passes, I shall see that the night itself only unveils new worlds of light; and to know that if I could unwrap fold after fold of God's universe, I should only unfold more and more blessing, and see deeper and deeper into the love which is at the heart of all.—*Elizabeth Charles.*

There is a winter more dreary than any mere season of Nature. The winter of sickness; long, painful days when it is well-nigh impossible to believe that health can ever resume its sway.

But health and strength return to us. We begin to feel the old joy in living, and something of the old zest and enthusiasm at the idea of taking up our life work again We thank God from our very hearts that He has seen fit to allow us to remain for awhile in His beautiful world. Very sweet is the "healing" of health's spring.—*F. Hurrell.*

### Notes and Queries.

**The Editor is always willing to answer in this column, without any fee, all reasonable questions, as soon as possible.**
**But the following rules must be carefully observed:—**
1. **Every communication must be accompanied by the name and address of the writer.**
2. **The question must always bear upon nursing, directly or indirectly.**

**If an answer is required by letter a fee of half-a-crown must be enclosed with the note containing the inquiry.**

*A. N. S. R.*

(37) Would you kindly advise me what to do in the following matter: At the outbreak of the war, I applied to be enrolled in the A. N. S. R., but was then unfortunately unsuccessful. Seeing that many nurses are still being sent out, do you think there would be any harm if I renewed my application or must I consider the first decision final?—*Sister Patrick.*

It will do you no harm to try again.

*Deafness.*

(38) I shall be greatly obliged if you will inform me how often, and in what quantity carate of soda should be dropped in ear for deafness, and is it preferable to dissolve the soda in oil or water?—*Cartaret.*

We presume that "Carteret" refers to carbonate of soda. But should carbonate of soda be dropped in the ear? That is the first question to decide, and that can only be decided by a doctor after a careful examination of the ear. Of course if used the soda should be dissolved in water.

*Electricity and Sciatica.*

(39) If you know of any work on electricity as applied for the cure of sciatica, I should esteem it a particular favour if you would kindly give me the title of it and the publisher's name.—*C. B.*

There is nothing special about the application of electricity in the treatment of sciatica. Refer to general treatises on medical uses of electricity.

*Treatment.*

(40) Would you let me know through the medium of your valuable paper the best treatment of membranous colitis and the duration of same and oblige.—*Nurse M.*

This depends on the case and must be decided by the practitioner in attendance.

*Aseptic Pus*

(41) Will you kindly tell me if there be such a thing as aseptic pus. For instance pus from the chest, "Empyema," will that be aseptic pus?—*Ignoramus.*

Yes. Pus may be aseptic, but the place it comes from will not decide the question. Empyema is sometimes very septic indeed.

*The Removal of Superfluous Hairs.*

(42) In THE HOSPITAL issued on February 23rd, there is an article describing the removal of superfluous hairs by the means of X-rays. I am anxious to know if there is any hospital in London where this operation is performed. Will you kindly give me the necessary information. Perhaps you can also tell me where Dr. Pusey practises.—*F. C.*

At any hospital which is properly equipped with X-ray apparatus, the removal of superfluous hairs by means of these rays can be done. But, of course, the medical officer in attendance must decide in any given case whether they should be removed, and what means should be used for the purpose. The paragraph referred to was an abstract from an article which appeared in the journal for cutaneous and genito-urine diseases, an American publication, and Dr. Pusey appears to be an American practitioner.

*Cancer Cure.*

(43) I have a relation who for some years past has undergone operations for cancer in the breast. She is now 45, and has had three separate operations. At present she has no open wound, and it is over a year since she had one. But all about her person small hard cancerous lumps form, and cause her much suffering. By her own and her friends' desire she is kept much under the influence of morphia, which seems quite to have destroyed her nerves, and she is in great misery. She thinks she has only a very few months to live. I think, and I believe the doctor feels the same, that life may be prolonged indefinitely. Perhaps she might be induced to take an interest in a cancer cure if it could be presented to her definitely. She is a clever good woman, but very wilful. She now lives far away from any railway station, in the country, with two professional nurses in attendance upon her, and no friend near to her. I want to put cases of cancer cure before her in an attractive light, can you help me? I should be grateful.

What is the name of a good doctor who might undertake it?

Are there good nursing homes in the neighbourhood of London for such a case? A garden and flowers desirable.—*E. C.*

It is difficult to understand what the writer means by a "cancer cure." The "cure" of a cancer, as of any other disease, depends upon the treatment, and that is a matter for the doctor. We cannot recommend a practitioner. There are plenty of nursing homes in London where any such case could be received, but whether it would be wise to remove such a patient from her home surroundings is another matter. The questions asked are such as can only be properly and usefully answered by the medical man who knows the patient, and has watched the progress of the case.

**Standard Books of Reference.**

"The Nursing Profession: How and Where to Train." 2s. net; post free 2s. 4d.
"The Nurses' Dictionary of Medical Terms." 2s.
"Burdett's Series of Nursing Text-Books." 1s. each.
"A Handbook for Nurses." (Illustrated.) 5s.
"Nursing: Its Theory and Practice." New Edition. 3s. 6d.
"Helps in Sickness and to Health." Fifteenth Thousand. 5s.
"The Physiological Feeding of Infants." 1s.
"The Physiological Nursery Chart." 1s.; post free, 1s. 3d.
"Hospital Expenditure: The Commissariat." 2s. 6d.

All these are published by THE SCIENTIFIC PRESS, Ltd., and may be obtained through any bookseller or direct from the publishers, 28 and 29 Southampton Street, London, W.C.

